# Lived experiences of mental health stigma and help-seeking among young Barbadians living with mental health conditions: A qualitative study

**DOI:** 10.1017/gmh.2026.10239

**Published:** 2026-06-02

**Authors:** Jay-Bethenny Gallimore, Maisha Holder, Donna-Maria Maynard, Graham Thornicroft, Petra C Gronholm, Tatiana Taylor Salisbury

**Affiliations:** 1 Centre for Global Mental Health, Health Service and Population Research Department, Institute of Psychiatry, King’s College London, UK; 2 Independent scholar; 3 Department of History, Philosophy and Psychology, Faculty of Humanities and Education, The University of the West Indies Cave Hill Campus, Barbados; 4 Centre for Global Mental Health, Department of Population Health, London School of Hygiene & Tropical Medicine, UK

**Keywords:** culture, global mental health, mental health, mental conditions, stigmatisation

## Abstract

In recent years, there has been increased public concern surrounding the mental health of young people in Barbados. A significant challenge for young people experiencing mental health conditions is stigma, which is a key barrier to help-seeking. This study aimed to explore how young Barbadians with mental health conditions experience and respond to mental health stigma and help-seeking. Qualitative semi-structured interviews were conducted in 2022 with 28 Barbadians aged 18–24 years who reported being diagnosed with a mental health condition, or self-identifying as having experienced mental health challenges. The data were analysed through reflexive thematic analysis. The findings highlighted the importance of culture in how mental health is perceived, and the role of anticipated and experienced stigma deterring help-seeking. Social networks acted as both facilitators and barriers to stigma and help-seeking, and mixed experiences and opinions of formal services raised concerns regarding the quality of care and accessibility. These findings highlight the need for locally informed, culturally relevant strategies and interventions to address and reduce stigma, facilitate help-seeking and improve care outcomes within the community in Barbados.

## Impact statement

Mental health within the Caribbean is a significant, yet long-neglected issue and remains greatly underrepresented in the global mental health literature. There is considerable historical and ongoing mental health stigma in the Caribbean. This hinders the understanding of this topic within the cultural context and the development of relevant interventions. In this qualitative study, we explored the lived experiences of mental health stigma and help-seeking among young people in Barbados living with mental health conditions. The findings highlight the integral role of culture and social networks in mental health perspectives, understandings and experiences, as well as the negative impact of anticipated and experienced stigma on help-seeking. Furthermore, opinions and experiences of formal health care point to the need for greater community care and improved quality and accessibility of services. This study contributes to understanding the significant burden mental health stigma places on well-being and how it acts as a pervasive barrier to help-seeking, especially in young people. It provides locally grounded, novel insights, which can serve as a valuable resource to support practice, policy and research implications within the community in Barbados.

## Introduction

It is estimated that at least 1 in 10 children and young people globally experience mental health challenges (Kaushik et al., 2016), with 50% of these challenges starting by age 14 and 75% by age 24 (Kessler et al., 2005). Untreated mental ill health in young people has been found to continue into adulthood, supporting the essential need for early identification and intervention (Radez et al., 2021). Poor mental health among young people can negatively impact emotions, behaviours, relationships, physical health, education, employment and quality of life, and can increase the risk of suicide (Dattani et al., 2018; Moitra et al., 2021). Although evidence-based mental health interventions are available (Reynolds et al., 2012), there are many reasons why young people often do not receive the care they need, including difficulty detecting mental health conditions and limited availability of services (Gulliver et al., 2010). Notably, one of the leading contributors to the exacerbation of mental health challenges and delays in seeking and accessing mental health treatment is stigma.

Mental health stigma can be defined as a problem of knowledge (i.e., a lack of or ignorance), negative attitudes (i.e., prejudice) and negative behaviour from stereotypes, misunderstandings or prejudice (i.e., discrimination) towards people with mental health conditions (Thornicroft et al., 2007). Literature has identified many types of stigma, including public stigma, internalised stigma, stigma by association and structural discrimination (Thornicroft et al., 2022). Public stigma refers to society’s beliefs and reactions towards those with mental health conditions. Internalised stigma, also known as self-stigma, references the internalisation of negative attitudes or beliefs commonly held by others. Stigma by association occurs due to one’s association with someone who is stigmatised. Structural discrimination describes the discrimination in laws, policies and practices. Individuals may also experience intersectional stigma, whereby multiple aspects of one’s identity are stigmatised, which converge, leading to unique experiences of disadvantage (Butler et al., 2025).

Stigma can be greatly shaped by cultural influences, which are collectively referred to as cultural stigma. Mental health has historically been a taboo topic in the Caribbean, with stigma remaining deeply embedded in cultural, economic, political and societal contexts (Doshi, 2017). Traditionally, poor mental health and expressing emotions have been culturally and socially stigmatised, associated with shame, personal weakness and a lack of commitment to a religious belief system (Bradshaw Maynard, 2013; Greenidge, 2016; Lacey et al., 2016). In Jamaica, stigma was identified as a key barrier to mental health help-seeking among young people (Maloney et al., 2020). A study conducted in Puerto Rico explored the experiences of stigma for those with Borderline Personality Disorder and identified that participants perceived a lack of understanding within society, as well as experiencing judgement and feelings of embarrassment from family and partners (Rivera-Segarra, 2014). Youssef et al. (2014) found that among Caribbean college students across Jamaica, Barbados and Trinidad and Tobago, mental health literacy was low, and poor attitudes towards mental health conditions were prevalent. These factors converge to create harmful narratives about mental health that reinforce stigma, creating barriers to open conversations, support and help-seeking.

Help-seeking can be categorised in many ways, including formal, informal and self-help (Rickwood et al., 2012). Research suggests that young people often prefer utilising informal sources of support (Cohen Goldstein et al., 2024). Jackson Williams (2012) found that Jamaican youth were most inclined to turn to family and friends regarding mental health challenges, although they remained sceptical concerning its efficacy in alleviating distress. Informal help-seeking typically precedes engagement with formal professional services (Yamasaki et al., 2016). However, a recent meta-synthesis on adolescent psychological help-seeking revealed that trust heavily influenced choices of disclosure, with concerns over confidentiality for both formal and informal sources (Barnes, 2019). This can contribute to the utilisation of self-help. Many young people have chosen the route of self-reliance, either due to personal choice, experienced or perceived stigma or lack of access to other forms of support, which can deter future interpersonal help-seeking (Ishikawa et al., 2023).

While the twenty-first century has seen an increase in mental health stigma research in the Caribbean (Mascayano et al., 2016), it remains a relatively under-researched area. A large proportion of the research conducted on mental health stigma and help-seeking within the Caribbean population has been conducted in settings outside of the Caribbean region (Gallimore et al., 2023). These findings cannot be generalised to Caribbean countries, which have their own unique colonial histories, cultural customs and norms, beliefs and healthcare systems. Additionally, the Caribbean is a diverse region, from high- to low-income countries, many languages, diverse social and cultural dynamics, ethnicities and political histories. Understanding how mental health stigma operates and impacts help-seeking, therefore, benefits from country-specific examination.

When considering Barbados specifically, in recent years, there has been increased public concern surrounding the mental health of young people (Barbados Today, 2025). Currently, mental health care is centralised to hospital-based care with limited community-based services, hindering the access and quality of care for individuals in need. While stigma likely contributes to the worsening of mental health, it remains unclear how stigma operates in the general population, limiting the country’s ability to develop evidence-based, locally tailored strategies for stigma reduction and to improve help-seeking, thus facilitating greater mental health outcomes.

Considering young people’s personal narratives of stigma and help-seeking is integral to understanding their opinions, perceptions and lived experiences. This approach allows their voices to be heard and informs the development of relevant programmes and policies suited to the Barbadian context. To our knowledge, this is the first study to explore how young Barbadians with mental health conditions experience and respond to mental health stigma and help-seeking. To achieve this aim, this study sought to address the research question: What are the lived experiences of mental health stigma and help-seeking among young people in Barbados?

## Methods

### Study design and setting

This study employed an exploratory qualitative approach, using individual semi-structured interviews to gather insights into participants’ opinions, perceptions and lived experiences regarding mental health stigma and help-seeking among Barbadian youth. Interviews were primarily conducted in person at the University of West Indies, Cave Hill Campus, a central location in Barbados. For participants unable to travel to the university, an appropriate alternative location was agreed upon, or the interview was conducted remotely via Zoom. All interviews were conducted in October and November 2022. The study is reported according to the Consolidated criteria for reporting qualitative research (COREQ) guidelines (Tong et al., 2007).

### Participant selection

Purposive sampling was used to identify participants meeting the inclusion criteria: aged 18–24 years, living in Barbados and diagnosed with a mental health condition or self-identifying as having experienced mental health challenges. Those who did not meet these criteria were excluded. Considering the high level of stigma present in Barbados, the inclusion of those without official diagnoses allowed for insights to be gathered from young people who may have avoided engaging with mental health services, allowing for a diverse range of experiences to be explored. A digital recruitment poster was shared with students in educational institutions by academics and with young people seeking mental health support by a local Counselling Psychologist. This poster, alongside information about the lead researcher, J-BG, was promoted on social media. J-BG also discussed the study on the radio to engage potential participants, and snowball sampling was employed, whereby existing participants would inform other potentially eligible individuals about the research. The target sample size was between 20 and 30, primarily guided by time constraints. However, there was no upper or lower limit defined. While data saturation is often utilised in qualitative research to determine sample size, this concept does not align with reflexive thematic analysis, the analytical method for this study (Braun and Clarke 2019). This approach asserts that saturation is unattainable as it is always possible for new insights to be generated. Therefore, JB-G conducted as many interviews as possible within the time and feasibility constraints.

### Procedures

The interviews were guided by a semi-structured topic guide (see Appendix S1), influenced by findings from a systematic review conducted by Gallimore et al. (2023) and discussions with all co-authors. Potential participants were given information about the study and data collection, and were provided in advance with the consent form. Socio-demographic information was gathered at the start of the interviews using open-ended questions that were subsequently coded. The interview procedure was piloted with one participant to test its feasibility. No revisions to questions or interview style were required, and subsequently, the data captured were integrated into the larger dataset. Interviews were conducted by J-BG and lasted ~1 h. Participants were debriefed at the end of their interviews and reminded of the list of support services provided within their participant information sheet. The interviews were audio-recorded and uploaded to Otter.ai for transcription. Each participant received $50 BBD (~20 GBP) as a token of appreciation for their time. This incentive aligned with local research procedures.

### Data analysis

Reflexive thematic analysis (TA) (Braun and Clarke, 2006) was the chosen method of data analysis. This method aligned with the reflexive approach that the lead author (J-BG) took when conducting the study. J-BG maintained a reflexivity journal throughout the data collection and analysis to continuously reflect on one’s own thoughts, assumptions and biases, and in turn assess how these may influence the interpretation of the data. The analysis was conducted inductively to allow for insights to be derived directly from the data, aligning with the exploratory research design adopted for this study (Stebbins, 2001). Following the six-step reflexive TA process, in Step 1, J-BG familiarised herself with the data by initially listening to all of the interviews using the transcription software Otter.ai (www.otter.ai), before re-listening a further three times, conducting transcription accuracy checks against the audio recording, anonymising the transcripts and making notes to begin identifying initial trends and interesting insights. All interviews were then transposed to Nvivo (Release 1.7.1) for Step 2, which involved the generation of initial codes, where the transcripts were reviewed line by line and codes applied to capture meaning data. Both semantic, capturing explicit meaning, and latent codes, focusing on deeper conceptual meanings, were created, refined and compiled into a list. In Step 3, codes were organised and clustered into groups of similar meanings to develop themes and subthemes. All themes were iteratively developed in Step 4, going back and forth between codes to ensure alignment with the themes and for the themes to cohesively tell a story of the analysis. Co-authors PCG and TTS assisted in this process. When a final set of themes had been established, a thematic map was created to visually illustrate the themes, subthemes and their interconnections. Step 5 involved refining, defining and naming the themes and subthemes. Direct anonymised participant quotations were attached to the themes and subthemes to illustrate their connection to the data. Lastly, Step 6 involved writing up the analysis as a narrative. Themes, subthemes and their interconnections continued to be refined during this final stage as insights and understanding of participants’ experiences developed, and quotations were reviewed between J-BG, PCG and TTS.

## Results

A total of 28 interviews were completed, with 24 interviews conducted in-person and the remaining four virtually. Interview duration ranged from 40 to 80 min. Participants reported a variety of mental health challenges, including depression, anxiety, bipolar disorder, schizophrenia, eating disorders and post-traumatic stress disorder. The demographic characteristics of the participants are shown in Table 1.

**Table 1.** Participant characteristics Table 1. long description.

Socio-demographic characteristics *n* = 28			
Variable	Subgroup	*n*	%
**Gender**	Male	6	21.4
	Female	20	71.4
	Non-binary	2	7.1
**Education**	Secondary school or less	14	50.0
	CAPE (Caribbean Advanced Proficiency Examinations)	4	14.3
	Tertiary	10	35.7
**Ethnicity**	Black/Caribbean/African American	23	82.1
	Indian	2	7.1
	Mixed	2	7.1
	Other	1	3.6
**Religion**	Christian	12	42.9
	Jehovah’s Witness	1	3.6
	Muslim	1	3.6
	None	8	28.6
	Other	6	21.4

Note: The socio-demographic characteristics captured, that is, Gender, Education, Ethnicity and Religion, of the 28 respondents are shown.

**Figure 1.**
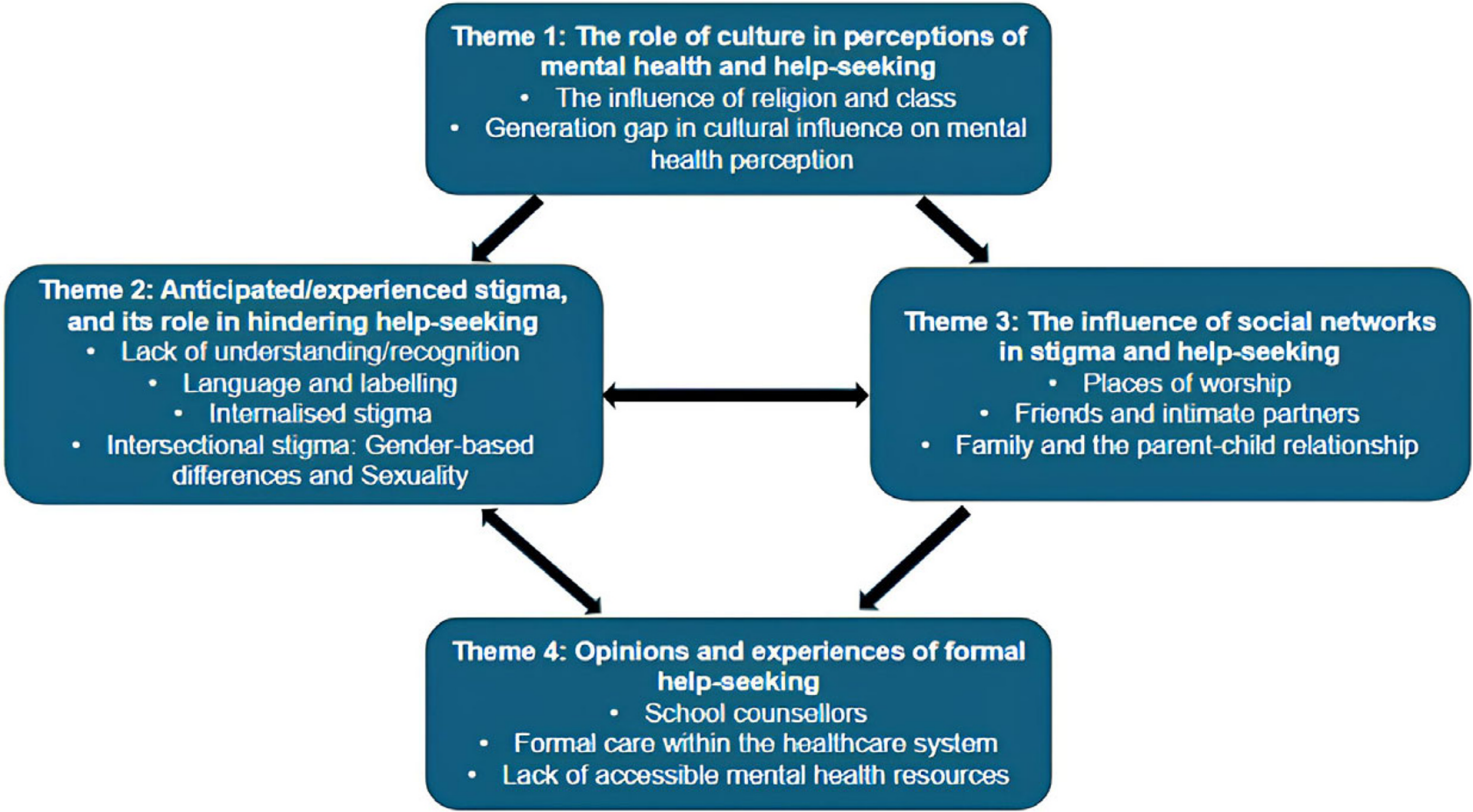
Thematic map of themes and subthemes. *Note:* Four themes and their respective subthemes are shown. The connections between each theme are illustrated through single-headed and double-headed arrows. Figure 1. long description.

The findings are presented under four key themes: (i) the role of culture in perceptions of mental health and help-seeking, (ii) anticipated/experienced stigma and its role in hindering help-seeking, (iii) the role of social networks in stigma and help-seeking and (iv) opinions and experiences of formal help-seeking. The 4 themes and their 12 subthemes are presented below in Figure 1.

### The role of culture in perceptions of mental health and help-seeking

Culture was identified to shape people’s perceptions and understanding of mental health and its related challenges, which influenced attitudes towards seeking help.

#### The influence of religion and class

Participants frequently expressed the influence of religion embedded within Barbadian culture, predominantly Christianity, as a contributor to stigma towards mental health challenges, leading to a lack of empathy and compassion.

*“Because my grandmother to this day, she’ll always say, ‘You need to come to church, you need to come to church and pray out that devil, that spirit of depression.’ And I just say “Grandma, it really is not necessarily that I have a demon, it’s my feelings.”* (Female, 21 years)

Some participants also commented on how religious-based beliefs of mental health contributed as a barrier to appropriate help-seeking support.

*“…the [mental health] advice may not be fully accurate…we advance so much that there’s so many new issues going on that the principles are still good, but there’s certain issues that you just can’t pick and choose where to go in the Bible.”* (Female, 20 years)

Classism was also raised as an issue within Barbadian culture, whereby mental health challenges are commonly perceived to be an issue experienced by the lower class, vulnerable communities in society.

*“…mental health is only for those mad people. For those crazy people, for the vulnerable [poor] people within our society.”* (Male, 23 years)

#### Generation gap in cultural influence on mental health perception

Participants discussed how older members of society often rejected notions of mental health, adhering to the Caribbean cultural norm of navigating hardship by ‘toughing it out’. Therefore, the challenges of young people were perceived to be insignificant and privileged compared to those faced by previous generations and therefore disregarded.

*“Speaking about mental issues in the Caribbean is generally a no because you got a roof over your head and you got clothes on your back. So how could you be sad?…they just see it as being ungrateful.”* (Female, 20 years)

While participants agreed that not all young people are understanding or compassionate towards mental health, there was an agreement that the younger generation is leading the shift in the cultural narrative around mental health to create a positive change.

*“From persons in my age group talking about it more…realising that the people before us, they went through the same thing or maybe worse…until it’s addressed or someone tries to fix it…, that can just keep happening again.”* (Female, 20 years)

### Anticipated/experienced stigma and its role in hindering help-seeking

The anticipation and experience of stigma created a notable barrier to seeking help and increased the risk of discrimination.

#### Lack of understanding/recognition

Participants reported that they often did not understand that what they were experiencing was mental health challenges, or that the seriousness was not recognised, and raised this as a common issue for many young people in society.

*“I feel like a lot of young children don’t know what’s going on with them so because they don’t talk about it, they’re just left in that stage and people are carrying on with their lives and not noticing oh that child, they might have some mental problem.*” (Female, 21 years)

Denial was frequently mentioned as a contributor to the reluctance in seeking help.

*“I think it’s [therapy] pointless at this point. Logically I know it would probably still help, but there’s probably some kind of denial at this point…like ‘I’m fine’.”* (Non-binary, 21 years)

#### Language and labelling

Negative language and labels attached to people with mental health conditions, such as ‘crazy’ and ‘mad’ were identified to perpetuate and exacerbate stigma and reduce the likelihood of help-seeking.

*“They say you’re mad, or you crazy or something wrong with you….It’s not good as it’s an illness that can be treated with the right kind of surroundings or environment around you.*” (Female, 21 years)

It was identified that more stigmatising language tended to be applied to severe mental health conditions, such as schizophrenia and bipolar disorder, compared to common mental health conditions, such as depression.

*“Bipolar, schizophrenia, people think you crazy. You would kill somebody. People depression be like, ‘Oh this a normal thing man. Everybody get depressed sometimes’.”* (Female, 23 years)

#### Internalised stigma

Many participants reported believing and accepting negative societal attitudes and beliefs about themselves, such as being ‘soft’, ‘weak’ or ‘crazy’. This internalisation often led to feelings of disappointment and insecurity.

*“I was telling myself I just being ungrateful, just being soft or weak. So it also manifested internally in myself, instead of maybe giving myself the grace that I needed in order to get through whatever trying time that I had.”* (Female, 20 years)

#### Intersectional stigma: Gender-based differences and sexuality

Many participants highlighted the prevalent stigma that surrounds men’s mental health, where traditional masculinity norms associate showing emotion as a weakness. Participants identified such emotional suppression as contributing to the violent behaviours and the increasing number of suicides identified across young male Barbadians.

*“If it’s suicide in Barbados it’s mostly a male. And then you still have males killing each other and stuff. And I think it’s just, us not being able to deal with our mental health issues or deal with our emotions in a positive way.”* (Male, 23 years)

Participants also raised the stigma surrounding sexual identity and sexual orientations outside of heterosexuality in Barbadian society, contributing to a reluctance to seek mental health support out of fear of further judgement.

*“When it comes to my gender and my sexuality…at that time no, I did not feel like I could talk to anyone about it because that also was something that was pushed under the rug.”* (Female, 21 years)

### The role of social networks in stigma and help-seeking

Social networks were identified to be both a valuable facilitator in reducing stigma and providing support, as well as a contributor to stigma and a barrier to help-seeking.

#### Places of worship

Many participants recalled the church associating mental health challenges with demonic possession, which led to a reluctance, or complete cessation, to confide in the church for support.

*“It was always you have a demon, you need to come and get prayed for…I was heavily in the church and when I was going to church, I wasn’t feeling any better…I felt like people in the church were letting me down.”* (Female, 21 years)

One participant, identifying as a Jehovah’s Witness, reported highly positive experiences from the ‘elders’ in seeking help and providing support.

*“When I go to the Kingdom Hall, I don’t worry about nothing. That’s the happiest place I could be…we call the elders if you have a problem… they going to answer even if they miss a call and call you back and text you later to make sure you are okay.”* (Female, 21 years)

Many participants highlighted how their personal relationships with God and spirituality were a positive source of mental health support.

*“I really do believe that spirituality and that personal relationship with God was one of the factors that played in getting me better, or me getting to this point in life.”* (Male, 23 years)

#### Friends and intimate partners

The connections of friends and intimate partners were often described as a positive source of comfort and help-seeking. However, this was also expressed with the caveat that these relationships can be an unhealthy substitute for professional help, whereby no one has the skills to provide the mental health support that is required.

*“…you have your friends that you could talk to, you can relate to. At the same time it’s like the blind leading the blind. So it’s not really helpful at the end of the day.”* (Female, 19 years)

#### Family and the parent–child relationship

Participants had varying experiences with family in relation to stigma and help-seeking. Most participants expressed a lack of support, where families were either unable or did not wish to recognise the challenges they were experiencing.

*“Some of my family members, they don’t want to believe that I have a mental problem. They would say, ‘Oh, man there ain’t nothing is wrong with you’…I feel annoyed because I know I have a problem.”* (Female, 23 years)

The parent–child relationship was the most important familial relationship in relation to acting as a barrier or facilitator of stigma and help-seeking. Most participants described an upbringing where they experienced abuse and neglect in the home, contributing to a reluctance to confide in their parents.

*“I don’t think the kids recognise signs of mental illness. I don’t think they recognize signs of mental abuse even…This starts literally from three, four years old…then they’re in adulthood and not fully functioning adults…and it’s sad to say, but it happens in the home…we think the abuse is normal.*” (Female, 24 years)

For the few participants who recounted loving, supportive relationships with their parents, this often reduced the shame they internalised regarding their mental health challenges.

*“…the way my mother is supportive, I don’t feel ashamed of it anymore. But my other friends that their parents are not so supportive, I can tell that they still have an element of shame about it.”* (Female, 19 years)

### Opinions and experiences of formal help-seeking

This theme captured participants’ opinions and experiences with a wide range of formal help-seeking, including inpatient treatment, counselling/therapy and medication.

#### School counsellors

The majority of participants recalled having school guidance counsellors during secondary school; however, many expressed being unaware of what type of support was available, and the infrequency of seeing guidance counsellors were barriers to help-seeking.

*“…guidance counsellors can also act has empowerment…letting children know that there’s a safe space, letting the children know that the services are available. And I think our education system lacks that.”* (Male, 23 years)

While some participants described positive experiences with school and university counsellors, overall, there was a lack of trust due to confidentiality breaches, which often led to a reluctance to seek further support from other help-seeking sources.

*“The guidance counsellor that I had…I felt that nothing I said to her was confidential. And I feel like I can’t trust people when I talk to them. So that’s why I’d rather not say anything.”* (Female, 21 years)

#### Formal care within the healthcare system

Three participants described experiences of inpatient care at the local hospital. All reported traumatic experiences that exacerbated their existing challenges and led to an avoidance of seeking further healthcare support.

*“I started harming myself after I left the hospital because I thought that it was so traumatic…I do not wish that on anybody…I haven’t been back to the hospital since.”* (Female, 21 years)

One participant experienced both inpatient and outpatient care at the psychiatric hospital and recalled their experience to be positive and integral to their recovery journey.

*“I think they do a good job with supporting patients, they’re very nice to them… It’s been good, [I’m] improving a lot.”* (Female, 23 years)

The stigma surrounding the psychiatric hospital led many participants to believe the care provided would be poor, contributing to a reluctance to speak out about their mental health challenges in fear of being admitted as a patient.

*“I don’t know if it’s true, but I’ve heard from the mental hospital that when you suicidal them is just leave you in a room, enclosed…So then I’m like, I don’t need to speak to my mental health or nobody, because I don’t need to end up down there.*” (Female, 23 years)

#### Lack of accessible mental health resources

Several participants discussed the restricted opening hours of the mental health hotline available in Barbados, which, at the time of the interviews, was 5–11 pm, 7 days a week. This was identified as a barrier to accessible help-seeking support during late hours of the night when many are in heightened mental health distress.

*“Our suicide hotline starts at 5pm and ends at 11pm, when statistically we know that the likelihood of suicides happens around midnight to about 5am. Why are you closed during those very important hours?”* (Female, 21 years)

Overall, many participants expressed the belief that there is a lack of local, accessible mental health resources to receive help. This was frequently cited to be due to perceived socio-economic barriers in society, including financial constraints and class.

*“I feel like Barbados has a lot of resources…but they don’t educate the people…we are very classist society…we only want certain resources to be accessible to certain classes of people.”* (Male, 23 years)

## Discussion

The current study sought to explore and understand how young Barbadians with mental health conditions experience and respond to stigma and help-seeking. The study identified cultural factors to play a significant role in stigma, where traditional religious views and classist narratives have often been a barrier to help-seeking. The findings reflected anticipated/experienced stigma to be a key deterrent to help-seeking. The importance of social networks, particularly the parent–child relationship, was identified, as was emphasis on care within formal care systems, whereby both improved access and quality of services within educational, healthcare and community settings can lessen stigma and reduce barriers to care.

Central to the study’s findings was the intricate role of culture in shaping experiences of stigma and help-seeking, particularly religion. Several participants found considerable benefit and support from their faith. However, many also experienced ostracisation and stigmatisation, due to their religious faith and community, namely Christianity and the Christian Church. This aligns with findings by Lloyd et al. (2023) who found that many Christian adults with mental health conditions reported positive experiences within the Christian community and religious system, such as community support and coping strategies. They also reported negative experiences stemming from stigma, exclusion and the spiritualisation of mental health challenges. Akadinma and Singh (2021) have highlighted the importance of collaboration between places of worship and mental health services to support the quality of care of individuals with mental health challenges with empathy and compassion.

Cultural norms regarding sexual identity and behaviour negatively impacted help-seeking among youth. Stigma associated with LGBTQ+ communities led to a reluctance to disclose mental health challenges. Additionally, societal expectations in Barbados for males, in particular, encourage emotional suppression. The socialisation of Caribbean males is one where showing emotion is an effeminate trait, and aggression and violence align with ‘what it means to be a man’ (Bailey and Coore-Desai, 2011). This subsequently negatively impacts their mental health and perpetuates resistance to help-seeking. These findings highlight the need to understand and challenge how socio-demographic factors such as sexuality and masculinity norms negatively impact mental health stigma, help-seeking and overall well-being for young people.

Poor experiences of mental health help-seeking, from both formal and informal sources, were a prominent deterrent to future treatment. These findings are congruent with the literature, highlighting positive and negative past experiences as a predictor of the likelihood of seeking help in young people (Radez et al., 2021). A particularly influential factor in the experiences of stigma and help-seeking was the parent–child relationship. Young people whose parents were nurturing, responsive and supportive were less likely to feel stigmatised by their parents for mental health challenges and more likely to seek help from formal and informal sources. This type of parenting aligns with characteristics of an authoritative parenting style (Baumrind, 1991). In this study, most participants described an upbringing experiencing physical and/or emotional abuse and manipulation, high parental demands and blame and shame, reflecting an authoritarian parenting style commonly adopted by many Caribbean parents (Baumrind, 1991; Lipps et al., 2012). This style of parenting has consistently been identified to contribute to poorer mental health, increased aggression and reduced help-seeking (Makwana et al., 2023; Yadav et al., 2021). These findings highlight the importance of the parent–child relationship in shaping children’s mental health and experiences of stigma and help-seeking.

Participants frequently expressed distrust of school counsellors as a deterrent to seeking psychological support, reporting accounts of experienced or known breaks in trust and privacy. In Jamaica, confidentiality has previously been cited as a reason as to why adolescents have resisted seeking help from guidance counsellors (Jackson Williams, 2012). Furthermore, the experiences of inpatient psychiatric hospitalisation within the local Barbados hospital echoed treatment reported by participants in previous studies globally– highly negative and traumatic, often with an increased risk of self-harming ideation and behaviours following hospitalisation (Walter et al., 2019). Together, the findings demonstrate how substantial improvements are needed within school and healthcare systems, including the training of care professionals to better support the mental health needs of young people in these settings.

Participants also expressed the need for mental health support during the late hours of the night, a time when research has shown that mental health distress and suicide ideation are heightened (Lok et al., 2024; Tubbs et al., 2021). In line with this preference, in 2024, Barbados launched a 24-h mental health hotline, which is expected to greatly facilitate accessible mental health service delivery (Rollock, 2024).

### Strengths and limitations

This is the first study to have explored the lived experiences of mental health stigma and help-seeking in young Barbadians. It addresses a significant knowledge gap in the literature and can serve as a foundation for future comparative studies across the Caribbean. The sample captured a diverse range of experiences of young Barbadians across mental health challenges, and various levels of engagement in formal and informal help-seeking. As a small subsection of the sample’s interviews were conducted via Zoom, this limited the collection of non-verbal cues, which assists in the interpretation of participants’ responses (Denham & Onwuegbuzie, 2013). However, the different modes of interview were not believed to have impacted the quality of the data and participants’ engagement in the conversation, which has similarly been the case in other studies (Anthony et al., 2025). The majority of participants identified as female, which may be reflective of the socialisation for men not to express their feelings, as identified in the findings of this study. This may impact the potential transferability of the findings to other contexts. Nevertheless, this study provides rich, contextual insights and understanding of this topic from a subset of the Barbadian youth population. This study also did not capture socio-demographic details such as household income or location by parish, which may have provided additional information regarding accessibility barriers and facilitators to care. Due to the small population of Barbados, the exclusion of collecting this data was determined to assist in preserving the anonymity of participants in a small island context.

### Implications for practice, policy and research

The findings of this study provide important considerations for the improvement of mental health care and research in Barbados. Mental health literacy should be actively incorporated into education from early years, positively exposing young people to the topic of mental health and well-being, which could lead to reduced levels of stigma and increase the likelihood of help-seeking. Counsellors, teachers and healthcare practitioners should receive cultural and trauma-informed mental health training to adequately support young people’s mental and emotional needs (Abrams, 2023). It is essential to engage parents and caregivers in efforts to reduce mental health stigma across the generations and facilitate support and access to care among young people (Smythe et al., 2024). Interventions and activities should be co-produced with young people and those with lived experience to support their relevance and applicability (Taylor Salisbury et al., 2021). There is an urgent need for legislative reform of Barbados’ Mental Health Act of 1985, which heavily focuses on institutional care. Jamaica has demonstrated the success of transitioning from institutional care to a community-based model, subsequently reducing public stigma (Hickling et al., 2011) and improving mental health outcomes (Hickling, 2021). This can serve as an example for adaptation to develop more culturally informed, humane care. Lastly, further Caribbean mental health research is needed in general to improve our understanding of the cultural and contextual factors that impact mental health and the development of interventions to reduce stigma and improve help-seeking and care.

## Conclusion

This study sought to explore and understand how young Barbadians with mental health conditions experience and respond to stigma and help-seeking. The insights gained from this study highlight the importance of developing targeted, culturally informed, youth-centred approaches that prioritise community-based approaches to stigma reduction and mental health care. Active collaborations between key stakeholders, including families, schools, healthcare professionals, policymakers and those with lived experience, can serve to reduce stigma, strengthen care systems, improve help-seeking and enhance mental health outcomes for young people in Barbados.

## Supporting information

10.1017/gmh.2026.10239.sm001 Gallimore et al. supplementary material Gallimore et al. supplementary material

## Data Availability

The authors have not made the transcripts publicly available; please contact the corresponding author for more information.

## Supplementary material

The supplementary material for this article can be found at http://doi.org/10.1017/gmh.2026.10239.

## Long descriptions

### Table 1. Long description

The socio-demographic characteristics captured, that is, Gender, Education, Ethnicity and Religion, of the 28 respondents are shown.

Navigate back to Table 1..

### Figure 1. Long description

Four themes and their respective subthemes are shown. The connections between each theme are illustrated through single-headed and double-headed arrows.

Navigate back to Figure 1..
