# Supplementary material for: Lived experiences of mental health stigma and help-seeking among young Barbadians living with mental health conditions: A qualitative study
Source: Glob Ment Health (Camb). 2026 Jun 2;13:e149. doi: 10.1017/gmh.2026.10239 (PMC13433337; doi:10.1017/gmh.2026.10239)
Supplement: Supplementary file 1 [file S2054425126102398sup001.docx]

# Appendices

Appendix S1

Interview Topic Guide

**About you**

- **Can we start by me asking you some questions about yourself so I can find out a little more about you?**

Prompts:

*How old are you and what you do for a living?/Are you a student?*

*What gender do you identify with?*

*How long have you lived in Barbados?  - Where were you born?*

*What ethnicity do you identify with?*

*Are you religious?*

*What is your highest level of education?*

*How did you find out about this study (recruitment route)?*

**Establishing mental health challenges**

- **Can you tell me a little bit about the mental health challenges you have experienced?**

Prompts:

*When did this start?*

*What were the first signs you noticed?*

*How did you make sense of what you were experiencing?*

*Did you have any ideas about what might be the problem?* Did you give a name to it yourself at all?

*Have you had a diagnosis of a condition?*

**Stigma**

- **What in your view do young people in Barbados think when they hear the phrase ‘mental illness’ or ‘mental health condition’?**

Why do you think this is?

- **Prior to your diagnosis/experiences with mental health challenges, what did you think of people with mental health challenges? Has that changed at all now?**

- (For those with a diagnosis) **Has your diagnosis changed how you feel about yourself?**/

(For those without a diagnosis) **Have your mental health challenges changed how you feel about yourself?**

**Does anyone know you experience mental health challenges?**

(If yes) **How has their reaction been? How do you feel about other people knowing?**

(If no) **How would you feel about other people knowing? What would make you feel this way?**

- **What do you think needs to happen to make sure that people with mental health challenges are not judged?**

**Help-seeking**

**So now we are going to talk about the support you may have sought**

- **Can you tell me about the first time you went and sort support?**

Prompts:

*Why did you go?*

*How did it feel for you to go and seek support?*

*Was it, planned or spontaneous?*

*What were your expectations? Hopes?*

*What actually happened?*

*What did you tell them?*

*What did they say back?*

*How did you feel afterwards?*

*Do you feel that it helped you?*

*What stopped you from attending X sooner?*

*What helped you to seek help from X at that time when you had not done so before?*

- **In what other ways have your sought support for your mental health challenges?**

Prompts:

*Has any particular experience stood out for you? For better or worse? Why?*

*What has influenced your decision of who to seek support from?*

*Confidentiality? Money? Ease of access?*

- **What has prevented you from seeking support?** (For participants who have not sought help)

Prompts:

*What do you think it would be like to tell someone?*

*Do you have any worries/concerns?*

*If you were in the future to see support, who do you think you would go to?*

*Why?*

*Who would you feel uncomfortable seeking support from?*

*Why?*

- **Do you have any coping strategies that you use to help you manage your mental health?**

- **What do you think about the support available for young people’s mental health in Barbados?**

Prompts:

*Do you think there is enough help available?*

*What do you think could be improved with Barbados mental health services?*

**Influence of demographics on mental health challenges, stigma experienced or help sought**

- **Gender, Religion, Ethnicity, Education (talk mental health in school, 11+), Family impact/understanding**

**Covid-19**

**I am now just going to ask a couple of questions linked to COVID-19.**

- **Have you felt that COVID-19 has had any impact on how mental health is viewed in Barbados?**

Prompts:

*Have you noticed more awareness of mental health since the pandemic?*

- **Do you feel that COVID-19 has impacted your mental health?**
- **Has COVID-19 had any impact on how you have sought help for your mental health challenges?**

**Reflections**

**We are coming to the end of our interview now.**

**Reflecting on your own experiences:**

- **What advice would you give to another young person in your community who is experiencing mental health challenges?**

**Closing**

- **And lastly, is there anything you else about your experiences you would like to mention?**

**Debrief**

**That is the end of our interview. Thank you so much for taking part. I am going to stop the recording now and we are just going to spend a little time talking about your experience of the interview.** **How are you feeling after the interview?**

- **How did you find the interview experience?**
- **Was it what you expected? If so, in what way?**
- **Were there any questions that made you feel uncomfortable?**
- **Is there anything you would have like to be asked about? Or questions you think would be good to add?**
- **Do you have any additional comments on how the interview process could be improved?**
- **On the participant information sheet I sent you, at the bottom there is a list of mental health resources if you feel you would like to access some additional support. You also have my email address if you have any further questions.**
