# Supplementary material for: Lived experiences of mental health stigma and help-seeking among young Barbadians living with mental health conditions: A qualitative study
Source: Glob Ment Health (Camb). 2026 Jun 2;13:e149. doi: 10.1017/gmh.2026.10239 (PMC13433337; doi:10.1017/gmh.2026.10239)
Supplement: Peer review history

# Reviewer report: Review: Lived experiences of mental health stigma and help-seeking among young Barbadians living with mental health conditions: A qualitative study — R0/PR2

**DOI:** 10.1017/gmh.2026.10239.pr2

This work makes a valuable contribution to understanding how different manifestations of mental health stigma influence young people’s decisions to seek mental health support and services. In addition to the presentation of the manuscript content, the study demonstrates conceptual and methodological consistency. The findings of this study have important implications for psychoeducation, mental health intervention, and community public health in Barbados.

Recommendations:

1. It would be important to include in the introduction literature from other Caribbean countries on stigma in mental health, in addition to that presented from Jamaica. For example, several qualitative studies in Puerto Rico have been conducted that address the stigma in mental health and its implications for seeking and adhering to treatment. This information could help expand knowledge about the manifestations of stigma that are inherent in the social and cultural factors of the Caribbean. You may consult the following articles: “That’s all Fake”: Health professionals’ stigma and physical healthcare of people living with Serious Mental Illness (https://doi.org/10.1371/journal.pone.0226401), Stigmatization Experiences among People Living with Borderline Personality Disorder in Puerto Rico (https://nsuworks.nova.edu/tqr/vol19/iss15/2/); and Stigma towards mental health problems among healthcare professionals in Puerto Rico (https://pepsic.bvsalud.org/pdf/salsoc/v4n3/4n3a03.pdf)

2. Participant selection

a. Include inclusion and exclusion criteria.

b. Consider the literature on “information power” that aligns with reflective thematic analysis to explain the rationale for the sample size. You may consult the article “Sample Size in Qualitative Interview Studies: Guided by Information Power” (https://doi.org/10.1177/1049732315617)

3. Procedures

a. Mention that sociodemographic information was collected from participants at the beginning of the interview, using open-ended questions that were then coded.

4. Data analysis

a. In the methodology section, the authors mention that the interview guide questions were developed based on a systematic review. It would be important to clarify whether the qualitative data were coded and analyzed considering areas or topics previously identified in the literature review. If so, authors should specify that a hybrid (deductive-inductive) logic was used during the data collection, codification, and analysis process.

5. Strengths and limitations

a. It would be important to know the authors' perspective on why there was greater participation by females than by males in the study.

b. I suggest removing the limitation on generalization and representativeness. As this is a qualitative study, these assumptions are not applicable. Authors could refer to the limitation of transferability. Authors may consult the article “Transferability and Generalization in Qualitative Research” (https://doi.org/10.1177/10497315241256560).

# Reviewer report: Review: Lived experiences of mental health stigma and help-seeking among young Barbadians living with mental health conditions: A qualitative study — R0/PR3

**DOI:** 10.1017/gmh.2026.10239.pr3

**Authors:** Benedict Weobong

**Affiliations:** School of Global Health, York University Faculty of Health, Canada

Lived experiences of mental health stigma and help-seeking among young Barbadians living with mental health conditions: a qualitative study.

General comments: 1

The paper should be considered for publication as it:

• Addresses and contributes to filling a critical gap in the literature on the process of stigmatization, particularly in a setting and demographic where little to no prior research has been done.

• The paper is well-written and easy to read. I would love to see this published and I’m sure many would read this paper with deep interest.

General comments: 2: Minor Essential Revisions

• Just a note to remind the authors to ensure they follow the COREQ guidelines in their report as this will extremely improve the structure/content of the paper and help with future systematic reviews in this subject area.

Title: the title as is, is perfect! Just wondering (and this is only a suggestion) if authors would want to make this a little bit catchy with a key quote from the data? I see several interesting quotes!

Specific comments:

This paper could be improved considerably if the following are addressed:

Abstract:

• Excellent!

Impact:

• Line 30: Clarification: it’s not clear how the study contributes to a significant burden….

Introduction:

• Excellent background articulating the public health significance of adolescent mental health particularly in the Caribbean and Barbados, the problem and why this study is important, and your approach to tackling the problem using a qualitative study design.

Methods:

General comments: Minor Essential Revisions

• Useful to state the specific qualitative study design employed. Was this phenomenology, grounded theory, ethnography etc?

• Under data analysis, useful to mention the software used in analysis (if any).

Results:

General comments: Minor Essential Revisions

• Might be useful to report distribution of mode of interviews – virtual, face-to-face.

• Wondering if authors were able to elicit stigma perpetuating factors?

• What about knowledge – was this a determinant of stigma? There’s good literature that suggests knowledge is not the central modifiable factor of the process of stigmatization. This reminds me: suggest authors use this terminology i.e. ‘process of stigmatization’ as there have been calls to ‘retire’ the use of the word ‘stigma’.

Discussion:

General comments: Minor Essential Revisions

• I really like the discussion. It was clear and nuanced.

• Regarding limitations, wonder if authors want to reflect on the different modes of interviews used and if this could potentially influence the findings given there’s a lot to pick-up in face-to-face qualitative interviews?

Weobong 2026

# Reviewer report: Review: Lived experiences of mental health stigma and help-seeking among young Barbadians living with mental health conditions: A qualitative study — R0/PR4

**DOI:** 10.1017/gmh.2026.10239.pr4

Abstract:

• Very clear abstract

• The distinction in terms of the type of stigma assessed is great

Impact statement:

• “This study contributes to a significant burden on wellbeing and acts as a pervasive barrier to help-seeking, especially in young people.”

o This sentence is not clear, what acts as the barrier?

Introduction:

• The paragraph on stigma is important and overall good, although I would distinguish between what is stigma versus what is discrimination and be clear about the relationship between stigma and discrimination as to not confuse readers

• Please define cultural stigma

• Great explanation as to the diversity of the Caribbean and why studying stigma in the specific settings, not just among populations is important

Methods:

• Detailing what recruitment material looked like and how people were engaged would be very helpful

• Did any IRB approve this study? If so, please detail that

Results:

• The results are well organized generally, however, more data as in quotes would comprehensively improve the paper, the descriptions of each results section are clear but then the data does not provide a full picture of what authors state is part of the thematic section

• For the influence of religion and culture, the first two quotes need more contextualization, it’s not clear the purpose of the quotes and how exactly they relate to mental health

• The lack of understanding/recognition section makes sense but I don’t see any quotes under the anticipated/experienced stigma section and the understanding/recognition does not belong here if it’s meant to be in this domain

• The labeling section makes sense for stigma but it should be clear it’s a subsection of the stigma domain

• The internalized and intersectional stigma sections are good but these should be detailed as types of stigma identified in the outset of the paper

• With the school counselors section, you state: “The majority of participants recalled having school guidance counsellors during secondary school, however many expressed being unaware of what type of support was available, and an infrequency of seeing guidance counsellors, were barriers to help-seeking.

o Please include data as to the being unaware and infrequency

Discussion:

• Nice discussion section

• I would caution use of stating that participants were “across mental health challenges” in the limitations section as it’s not clear how you would know this, however if you do know this it would be good to include in the methods and results

# Editor report: Recommendation: Lived experiences of mental health stigma and help-seeking among young Barbadians living with mental health conditions: A qualitative study — R0/PR5

**DOI:** 10.1017/gmh.2026.10239.pr5

**Affiliations:** Ponce Health Sciences University, Puerto Rico

No accompanying comment.

# Editor report: Decision: Lived experiences of mental health stigma and help-seeking among young Barbadians living with mental health conditions: A qualitative study — R0/PR6

**DOI:** 10.1017/gmh.2026.10239.pr6

**Affiliations:** London School of Hygiene & Tropical Medicine, United Kingdom of Great Britain and Northern Ireland

No accompanying comment.

# Editor report: Recommendation: Lived experiences of mental health stigma and help-seeking among young Barbadians living with mental health conditions: A qualitative study — R1/PR8

**DOI:** 10.1017/gmh.2026.10239.pr8

**Affiliations:** Ponce Health Sciences University, Puerto Rico

No accompanying comment.

# Editor report: Decision: Lived experiences of mental health stigma and help-seeking among young Barbadians living with mental health conditions: A qualitative study — R1/PR9

**DOI:** 10.1017/gmh.2026.10239.pr9

**Affiliations:** London School of Hygiene & Tropical Medicine, United Kingdom of Great Britain and Northern Ireland

No accompanying comment.
